# Bis[2-(pyrrolidin-2-yl)-1*H*-benzimidazole-κ^2^ *N* ^2^,*N* ^3^]copper(II) dinitrate dihydrate

**DOI:** 10.1107/S160053680901808X

**Published:** 2009-05-20

**Authors:** Jing Dai

**Affiliations:** a Ordered Matter Science Research Center, College of Chemistry and Chemical Engineering, Southeast University, Nanjing 210096, People’s Republic of China

## Abstract

In the title compound, [Cu(C_11_H_13_N_3_)_2_](NO_3_)_2_·2H_2_O, synthesized by hydro­thermal reaction of Cu(NO_3_)_2_ and racemic 2-(pyrrolidin-2-yl)-1*H*-1,3-benzimidazole, the Cu^II^ atom lies on an inversion centre. The distorted octa­hedral Cu^II^ environment contains two planar *trans*-related *N*,*N*-chelating 2-(pyrrolidin-2-yl)-1*H*-1,3-benzimidazole ligands in the equatorial plane and two monodentate nitrate anions, which are in weak inter­action with the Cu atom, in the axial positions. The two benzimidazole ligands have opposite configurations (*R*/*S* and *S*/*R*) and compound is a *meso* complex. In the crystal, N—H⋯O and O—H⋯O hydrogen bonds generate an infinite three-dimensional network. One methylene group of the pyrrolidine ring is disordered over two position with a 0.56 (3):0.44 (3) occupancy.

## Related literature

For physical properties such as the ferroelectric and dielectric behavior of metal-organic coordination compounds, see: Fu *et al.* (2007 ▶). For the synthesis, see: Aminabhavi *et al.* (1986 ▶). For related structures, see: Dai & Fu (2008*a* ▶,*b* ▶); Fu & Ye (2007 ▶).
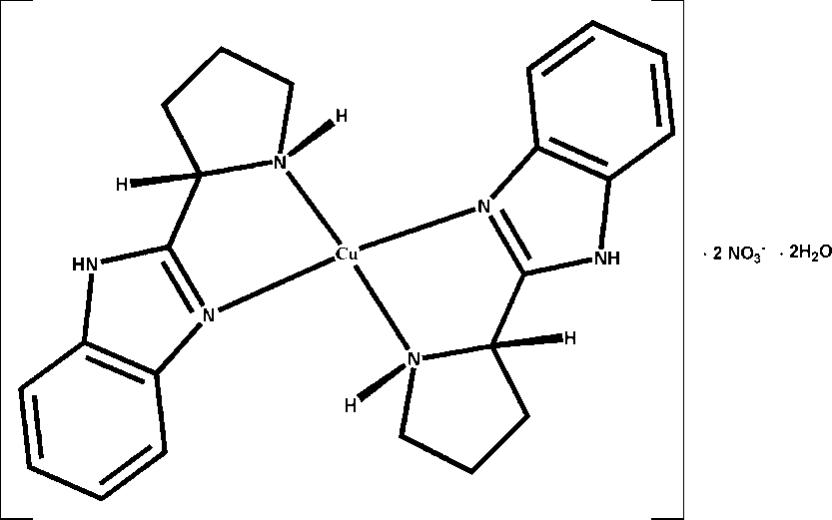


## Experimental

### 

#### Crystal data

[Cu(C_11_H_13_N_3_)_2_](NO_3_)_2_·2H_2_O
*M* *_r_* = 598.08
Triclinic, 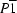

*a* = 8.2790 (17) Å
*b* = 8.4446 (17) Å
*c* = 9.759 (2) Å
α = 100.37 (3)°
β = 107.15 (3)°
γ = 91.37 (3)°
*V* = 639.1 (2) Å^3^
*Z* = 1
Mo *K*α radiation
μ = 0.92 mm^−1^
*T* = 298 K
0.35 × 0.30 × 0.15 mm

#### Data collection

Rigaku Mercury2 diffractometer
Absorption correction: multi-scan (*CrystalClear*; Rigaku, 2005 ▶) *T*_min_ = 0.732, *T*_max_ = 0.871
6713 measured reflections
2914 independent reflections
2566 reflections with *I* > 2σ(*I*)
*R*_int_ = 0.027

#### Refinement

*R*[*F* ^2^ > 2σ(*F* ^2^)] = 0.043
*wR*(*F* ^2^) = 0.101
*S* = 1.11
2914 reflections
188 parameters
6 restraints
H-atom parameters constrained
Δρ_max_ = 0.33 e Å^−3^
Δρ_min_ = −0.31 e Å^−3^

### 

Data collection: *CrystalClear* (Rigaku, 2005 ▶); cell refinement: *CrystalClear*; data reduction: *CrystalClear*; program(s) used to solve structure: *SHELXS97* (Sheldrick, 2008 ▶); program(s) used to refine structure: *SHELXL97* (Sheldrick, 2008 ▶); molecular graphics: *SHELXTL* (Sheldrick, 2008 ▶); software used to prepare material for publication: *SHELXTL*.

## Supplementary Material

Crystal structure: contains datablocks I, global. DOI: 10.1107/S160053680901808X/dn2443sup1.cif

Structure factors: contains datablocks I. DOI: 10.1107/S160053680901808X/dn2443Isup2.hkl

Additional supplementary materials:  crystallographic information; 3D view; checkCIF report

## Figures and Tables

**Table 1:** Hydrogen-bond geometry (Å, °)

*D*—H⋯*A*	*D*—H	H⋯*A*	*D*⋯*A*	*D*—H⋯*A*
N3—H3*B*⋯O1^i^	0.91	2.25	2.986 (3)	137
O1*W*—H1*WA*⋯O2^ii^	0.93	1.92	2.836 (4)	169
O1*W*—H1*WB*⋯O2^iii^	0.98	1.94	2.861 (3)	155
N2—H2*B*⋯O1*W*	0.86	1.86	2.706 (3)	168

Symmetry codes: (i) 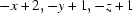; (ii) 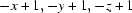; (iii) 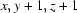.

**Fig. 1.**
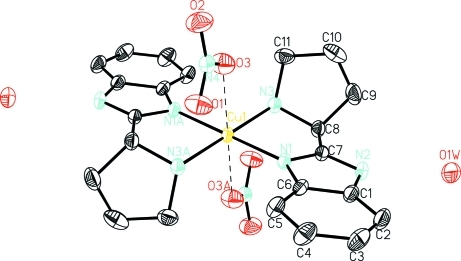
A view of the title compound with the atomic numbering scheme. Displacement ellipsoids were drawn at the 30% probability level. H atoms have been omitted. The weak interaction of CuII with nitrate is shown.

**Fig. 2.**
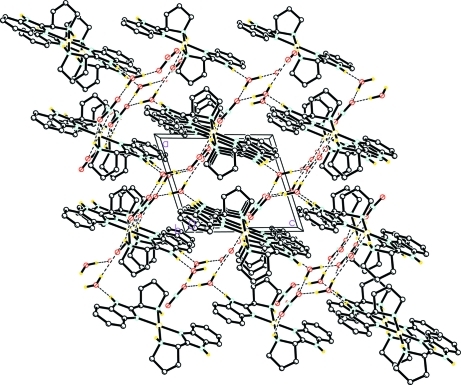
The crystal packing of the title compound viewed along the a axis and all hydrogen atoms not involved in hydrogen bonding (dashed lines) were omitted for clarity.

## supplementary crystallographic information

### Comment

Amino acid derivatives have found wide range of applications in material science, such as ferroelectric, fluorescence and dielectric behaviors. And there has been an increased interest in the preparation of amino acid coordination compound (Aminabhavi *et al., *1986; Dai & Fu 2008*a*; Fu & Ye 2007; Dai & Fu 2008*b*; Fu, *et al.* 2007). We report here the crystal structure of the title compound, [Nitrate-[2-(pyrrolidin-2-yl)-1*H*-benzimidazole] Copper(II)] dihydrate.

In the title compound, the Cu^II^ atom lies on an inversion centre. The distorted octahedral Cu^II^ environment contains two planar *trans*-related *N*,*N*-chelating 2-(pyrrolidin-2-yl)-1*H*-1,3-benzimidazole ligands in the equatorial plane and two monodentate nitrate anion ligands which are in weak interaction with the Cu atom in the axial position. The two benzimidazole ligands have opposite configuration *R*,*S* and S,*R* and the complex is *meso*(Fig. 1).

In the crystal structure, molecules are linked into a three-dimension network by N—H···O and O—H···O hydrogen bonds.(Fig.2, Table 1).

### Experimental

The racemic ligand 2-(pyrrolidin-2-yl)-1*H*-benzo[*d*]imidazole was synthesized by reaction of *S*-pyrrolidine-2-carboxylic acid and benzene-1,2-diamine according to the procedure described in the literature(Aminabhavi, *et al.*(1986)). A mixture of 2-(pyrrolidin-2-yl)-1*H*-benzo[*d*]imidazole (0.1 mmol) and Cu(NO_3_)_2_ (0.1 mmol) and water (1 ml) sealed in a glass tube were maintained at 70 °C. Crystals suitable for X-ray analysis were obtained after 5 days.

### Refinement

All H atoms attached to C and N atoms were fixed geometrically and treated as riding with C—H = 0.93 Å (aromatic), 0.97 Å (methylene) or 0.98 Å (methine) and N—H = 0.91 Å (N3), 0.86 Å (N2) with *U*_iso_(H) = 1.2*U*_eq_(C,*N*). H atoms of water molecule located in difference Fouriermaps and in the last stage of refinement they were treated as riding on the O atom with *U*_iso_(H) = 1.5*U*_eq_(O).

One of the pyrrolidine rings is disordered with the C10 atom statistically distributed over two positions. These disorders were treated using the tools (SAME and PART) available in *SHELXL97* (Sheldrick, 2008).

### Crystal data

**Table:**

[Cu(C_11_H_13_N_3_)_2_](NO_3_)_2_·2H_2_O	*Z* = 1
*M**_r_* = 598.08	*F*(000) = 311
Triclinic, *P*1	*D*_x_ = 1.554 Mg m^−^^3^
Hall symbol: -P 1	Mo *K*α radiation, λ = 0.71073 Å
*a* = 8.2790 (17) Å	Cell parameters from 2913 reflections
*b* = 8.4446 (17) Å	θ = 3.4–27.5°
*c* = 9.759 (2) Å	µ = 0.92 mm^−^^1^
α = 100.37 (3)°	*T* = 298 K
β = 107.15 (3)°	Block, blue
γ = 91.37 (3)°	0.35 × 0.30 × 0.15 mm
*V* = 639.1 (2) Å^3^	

### Data collection

**Table:**

Rigaku Mercury2 diffractometer	2914 independent reflections
Radiation source: fine-focus sealed tube	2566 reflections with *I* > 2σ(*I*)
graphite	*R*_int_ = 0.027
Detector resolution: 13.6612 pixels mm^-1^	θ_max_ = 27.5°, θ_min_ = 3.4°
CCD profile fitting scans	*h* = −10→10
Absorption correction: multi-scan (*CrystalClear*; Rigaku, 2005)	*k* = −10→10
*T*_min_ = 0.732, *T*_max_ = 0.871	*l* = −12→12
6713 measured reflections	

### Refinement

**Table:**

Refinement on *F*^2^	Primary atom site location: structure-invariant direct methods
Least-squares matrix: full	Secondary atom site location: difference Fourier map
*R*[*F*^2^ > 2σ(*F*^2^)] = 0.043	Hydrogen site location: inferred from neighbouring sites
*wR*(*F*^2^) = 0.101	H-atom parameters constrained
*S* = 1.11	*w* = 1/[σ^2^(*F*_o_^2^) + (0.0352*P*)^2^ + 0.444*P*] where *P* = (*F*_o_^2^ + 2*F*_c_^2^)/3
2914 reflections	(Δ/σ)_max_ < 0.001
188 parameters	Δρ_max_ = 0.33 e Å^−^^3^
6 restraints	Δρ_min_ = −0.31 e Å^−^^3^

### Special details

**Table:**

Geometry. All e.s.d.'s (except the e.s.d. in the dihedral angle between two l.s. planes) are estimated using the full covariance matrix. The cell e.s.d.'s are taken into account individually in the estimation of e.s.d.'s in distances, angles and torsion angles; correlations between e.s.d.'s in cell parameters are only used when they are defined by crystal symmetry. An approximate (isotropic) treatment of cell e.s.d.'s is used for estimating e.s.d.'s involving l.s. planes.
Refinement. Refinement of *F*^2^ against ALL reflections. The weighted *R*-factor *wR* and goodness of fit *S* are based on *F*^2^, conventional *R*-factors *R* are based on *F*, with *F* set to zero for negative *F*^2^. The threshold expression of *F*^2^ > σ(*F*^2^) is used only for calculating *R*-factors(gt) *etc*. and is not relevant to the choice of reflections for refinement. *R*-factors based on *F*^2^ are statistically about twice as large as those based on *F*, and *R*- factors based on ALL data will be even larger.

### Fractional atomic coordinates and isotropic or equivalent isotropic displacement parameters (Å^2^)

**Table:**

	*x*	*y*	*z*	*U*_iso_*/*U*_eq_	Occ. (<1)
Cu1	1.0000	0.5000	0.5000	0.03289 (14)	
O1	0.8537 (3)	0.0645 (3)	0.4119 (3)	0.0643 (6)	
O2	0.6487 (3)	0.0360 (3)	0.2118 (3)	0.0745 (7)	
O3	0.7605 (3)	0.2727 (3)	0.3222 (3)	0.0647 (6)	
N1	0.9102 (2)	0.4814 (2)	0.6652 (2)	0.0329 (4)	
N2	0.7676 (3)	0.5950 (3)	0.8117 (2)	0.0406 (5)	
H2B	0.7115	0.6647	0.8502	0.049*	
N3	0.8509 (3)	0.6877 (2)	0.4834 (2)	0.0345 (4)	
H3B	0.9103	0.7701	0.4663	0.041*	
N4	0.7551 (3)	0.1246 (3)	0.3161 (2)	0.0425 (5)	
C1	0.8109 (3)	0.4496 (3)	0.8525 (3)	0.0383 (6)	
C2	0.7753 (4)	0.3760 (4)	0.9580 (3)	0.0507 (7)	
H2A	0.7151	0.4256	1.0184	0.061*	
C3	0.8337 (4)	0.2263 (4)	0.9686 (3)	0.0552 (8)	
H3A	0.8115	0.1721	1.0369	0.066*	
C4	0.9250 (4)	0.1551 (4)	0.8794 (3)	0.0564 (8)	
H4A	0.9643	0.0545	0.8909	0.068*	
C5	0.9603 (4)	0.2272 (3)	0.7737 (3)	0.0467 (6)	
H5A	1.0208	0.1769	0.7139	0.056*	
C6	0.9014 (3)	0.3778 (3)	0.7609 (2)	0.0348 (5)	
C7	0.8291 (3)	0.6078 (3)	0.7009 (3)	0.0338 (5)	
C8	0.8131 (3)	0.7461 (3)	0.6234 (3)	0.0380 (6)	
H8A	0.8966	0.8350	0.6830	0.046*	
C9	0.6378 (4)	0.8083 (5)	0.5821 (4)	0.0658 (9)	
H9A	0.5789	0.7940	0.6520	0.079*	
H9B	0.6446	0.9216	0.5764	0.079*	
C11	0.6832 (4)	0.6571 (4)	0.3660 (3)	0.0542 (8)	
H11A	0.6811	0.7192	0.2911	0.065*	
H11B	0.6610	0.5435	0.3209	0.065*	
O1W	0.5901 (3)	0.7856 (3)	0.9583 (3)	0.0757 (7)	
H1WA	0.5045	0.8317	0.8968	0.113*	
H1WB	0.6169	0.8451	1.0596	0.113*	
C10	0.5552 (12)	0.709 (2)	0.4410 (13)	0.066 (3)	0.56 (3)
H10A	0.4736	0.7698	0.3822	0.079*	0.56 (3)
H10B	0.4948	0.6151	0.4524	0.079*	0.56 (3)
C10'	0.5655 (18)	0.773 (2)	0.4195 (16)	0.055 (3)	0.44 (3)
H10C	0.5642	0.8711	0.3809	0.066*	0.44 (3)
H10D	0.4507	0.7226	0.3896	0.066*	0.44 (3)

### Atomic displacement parameters (Å^2^)

**Table:**

	*U*^11^	*U*^22^	*U*^33^	*U*^12^	*U*^13^	*U*^23^
Cu1	0.0347 (2)	0.0409 (3)	0.0285 (2)	0.01231 (17)	0.01597 (17)	0.00874 (17)
O1	0.0575 (13)	0.0570 (13)	0.0686 (14)	0.0016 (10)	−0.0016 (11)	0.0231 (11)
O2	0.0782 (16)	0.0615 (14)	0.0594 (14)	0.0032 (12)	−0.0028 (13)	−0.0098 (11)
O3	0.0681 (15)	0.0449 (12)	0.0709 (15)	0.0090 (10)	0.0053 (12)	0.0119 (11)
N1	0.0356 (10)	0.0360 (10)	0.0300 (10)	0.0063 (8)	0.0148 (8)	0.0052 (8)
N2	0.0427 (12)	0.0473 (12)	0.0368 (11)	0.0108 (10)	0.0227 (10)	0.0027 (9)
N3	0.0368 (11)	0.0351 (10)	0.0355 (11)	0.0066 (8)	0.0161 (9)	0.0077 (8)
N4	0.0372 (12)	0.0490 (13)	0.0411 (12)	0.0057 (10)	0.0151 (10)	0.0025 (10)
C1	0.0386 (13)	0.0454 (14)	0.0311 (12)	−0.0013 (11)	0.0142 (10)	0.0025 (10)
C2	0.0549 (17)	0.0651 (19)	0.0374 (14)	−0.0001 (14)	0.0250 (13)	0.0056 (13)
C3	0.075 (2)	0.0581 (18)	0.0381 (15)	−0.0063 (16)	0.0241 (14)	0.0133 (13)
C4	0.085 (2)	0.0457 (16)	0.0441 (16)	0.0056 (15)	0.0246 (16)	0.0150 (13)
C5	0.0660 (18)	0.0421 (15)	0.0378 (14)	0.0090 (13)	0.0244 (13)	0.0076 (11)
C6	0.0401 (13)	0.0387 (13)	0.0257 (11)	0.0003 (10)	0.0127 (10)	0.0024 (10)
C7	0.0313 (12)	0.0393 (13)	0.0306 (12)	0.0026 (10)	0.0120 (10)	0.0016 (10)
C8	0.0420 (14)	0.0362 (13)	0.0383 (13)	0.0094 (11)	0.0177 (11)	0.0033 (10)
C9	0.064 (2)	0.081 (2)	0.071 (2)	0.0437 (18)	0.0356 (18)	0.0315 (19)
C11	0.0545 (18)	0.0480 (16)	0.0471 (16)	0.0155 (13)	−0.0014 (14)	0.0035 (13)
O1W	0.0743 (16)	0.0971 (18)	0.0527 (13)	0.0449 (14)	0.0207 (12)	0.0000 (12)
C10	0.036 (3)	0.071 (7)	0.086 (5)	0.000 (4)	0.003 (3)	0.032 (5)
C10'	0.041 (5)	0.049 (7)	0.071 (6)	0.018 (5)	0.012 (4)	0.009 (5)

### Geometric parameters (Å, °)

**Table:**

Cu1—N1^i^	1.9922 (19)	C4—C5	1.384 (4)
Cu1—N1	1.9922 (19)	C4—H4A	0.9300
Cu1—N3	2.032 (2)	C5—C6	1.386 (4)
Cu1—N3^i^	2.032 (2)	C5—H5A	0.9300
O1—N4	1.241 (3)	C7—C8	1.490 (3)
O2—N4	1.240 (3)	C8—C9	1.520 (4)
O3—N4	1.241 (3)	C8—H8A	0.9800
N1—C7	1.324 (3)	C9—C10	1.438 (13)
N1—C6	1.405 (3)	C9—C10'	1.492 (15)
N2—C7	1.343 (3)	C9—H9A	0.9700
N2—C1	1.382 (3)	C9—H9B	0.9700
N2—H2B	0.8600	C11—C10	1.488 (12)
N3—C8	1.491 (3)	C11—C10'	1.529 (11)
N3—C11	1.499 (3)	C11—H11A	0.9700
N3—H3B	0.9100	C11—H11B	0.9700
C1—C2	1.391 (4)	O1W—H1WA	0.9281
C1—C6	1.397 (3)	O1W—H1WB	0.9827
C2—C3	1.375 (4)	C10—H10A	0.9700
C2—H2A	0.9300	C10—H10B	0.9700
C3—C4	1.383 (4)	C10'—H10C	0.9700
C3—H3A	0.9300	C10'—H10D	0.9700
			
N1^i^—Cu1—N1	180.000 (1)	N1—C7—C8	121.5 (2)
N1^i^—Cu1—N3	97.34 (8)	N2—C7—C8	126.0 (2)
N1—Cu1—N3	82.66 (8)	C7—C8—N3	106.82 (19)
N1^i^—Cu1—N3^i^	82.66 (8)	C7—C8—C9	115.3 (2)
N1—Cu1—N3^i^	97.34 (8)	N3—C8—C9	106.4 (2)
N3—Cu1—N3^i^	180.00 (12)	C7—C8—H8A	109.4
C7—N1—C6	105.71 (19)	N3—C8—H8A	109.4
C7—N1—Cu1	112.43 (16)	C9—C8—H8A	109.4
C6—N1—Cu1	141.85 (16)	C10—C9—C8	102.8 (6)
C7—N2—C1	107.5 (2)	C10'—C9—C8	108.6 (5)
C7—N2—H2B	126.2	C10—C9—H9A	111.2
C1—N2—H2B	126.2	C10'—C9—H9A	126.7
C8—N3—C11	106.2 (2)	C8—C9—H9A	111.2
C8—N3—Cu1	110.88 (15)	C10—C9—H9B	111.2
C11—N3—Cu1	116.78 (16)	C10'—C9—H9B	87.2
C8—N3—H3B	107.5	C8—C9—H9B	111.2
C11—N3—H3B	107.5	H9A—C9—H9B	109.1
Cu1—N3—H3B	107.5	C10—C11—N3	105.4 (5)
O2—N4—O3	118.9 (2)	N3—C11—C10'	106.2 (5)
O2—N4—O1	119.9 (2)	C10—C11—H11A	110.7
O3—N4—O1	121.2 (2)	N3—C11—H11A	110.7
N2—C1—C2	131.1 (2)	C10'—C11—H11A	88.8
N2—C1—C6	106.1 (2)	C10—C11—H11B	110.7
C2—C1—C6	122.8 (3)	N3—C11—H11B	110.7
C3—C2—C1	116.6 (3)	C10'—C11—H11B	129.2
C3—C2—H2A	121.7	H11A—C11—H11B	108.8
C1—C2—H2A	121.7	H1WA—O1W—H1WB	110.4
C2—C3—C4	121.0 (3)	C9—C10—C11	109.9 (6)
C2—C3—H3A	119.5	C9—C10—H10A	109.7
C4—C3—H3A	119.5	C11—C10—H10A	109.7
C3—C4—C5	122.7 (3)	C9—C10—H10B	109.7
C3—C4—H4A	118.7	C11—C10—H10B	109.7
C5—C4—H4A	118.7	H10A—C10—H10B	108.2
C4—C5—C6	117.1 (3)	C9—C10'—C11	104.9 (8)
C4—C5—H5A	121.5	C9—C10'—H10C	110.8
C6—C5—H5A	121.5	C11—C10'—H10C	110.8
C5—C6—C1	119.8 (2)	C9—C10'—H10D	110.8
C5—C6—N1	132.0 (2)	C11—C10'—H10D	110.8
C1—C6—N1	108.1 (2)	H10C—C10'—H10D	108.8
N1—C7—N2	112.5 (2)		
			
N3—Cu1—N1—C7	−11.34 (17)	Cu1—N1—C7—C8	−0.8 (3)
N3^i^—Cu1—N1—C7	168.66 (17)	C1—N2—C7—N1	−0.1 (3)
N3—Cu1—N1—C6	168.4 (3)	C1—N2—C7—C8	−179.6 (2)
N3^i^—Cu1—N1—C6	−11.6 (3)	N1—C7—C8—N3	17.5 (3)
N1^i^—Cu1—N3—C8	−159.18 (16)	N2—C7—C8—N3	−163.0 (2)
N1—Cu1—N3—C8	20.82 (16)	N1—C7—C8—C9	135.5 (3)
N1^i^—Cu1—N3—C11	79.0 (2)	N2—C7—C8—C9	−45.1 (4)
N1—Cu1—N3—C11	−101.0 (2)	C11—N3—C8—C7	102.9 (2)
C7—N2—C1—C2	−178.2 (3)	Cu1—N3—C8—C7	−24.9 (2)
C7—N2—C1—C6	0.3 (3)	C11—N3—C8—C9	−20.7 (3)
N2—C1—C2—C3	178.1 (3)	Cu1—N3—C8—C9	−148.6 (2)
C6—C1—C2—C3	−0.2 (4)	C7—C8—C9—C10	−88.1 (7)
C1—C2—C3—C4	0.8 (5)	N3—C8—C9—C10	30.0 (7)
C2—C3—C4—C5	−1.2 (5)	C7—C8—C9—C10'	−112.9 (9)
C3—C4—C5—C6	0.7 (5)	N3—C8—C9—C10'	5.3 (10)
C4—C5—C6—C1	−0.1 (4)	C8—N3—C11—C10	3.6 (8)
C4—C5—C6—N1	−178.1 (3)	Cu1—N3—C11—C10	127.8 (7)
N2—C1—C6—C5	−178.9 (2)	C8—N3—C11—C10'	28.3 (10)
C2—C1—C6—C5	−0.2 (4)	Cu1—N3—C11—C10'	152.6 (9)
N2—C1—C6—N1	−0.4 (3)	C10'—C9—C10—C11	78.5 (18)
C2—C1—C6—N1	178.3 (2)	C8—C9—C10—C11	−28.4 (12)
C7—N1—C6—C5	178.6 (3)	N3—C11—C10—C9	16.2 (12)
Cu1—N1—C6—C5	−1.1 (5)	C10'—C11—C10—C9	−79 (2)
C7—N1—C6—C1	0.4 (3)	C10—C9—C10'—C11	−68.1 (16)
Cu1—N1—C6—C1	−179.4 (2)	C8—C9—C10'—C11	11.8 (14)
C6—N1—C7—N2	−0.2 (3)	C10—C11—C10'—C9	67 (2)
Cu1—N1—C7—N2	179.63 (16)	N3—C11—C10'—C9	−24.6 (14)
C6—N1—C7—C8	179.4 (2)		

Symmetry codes: (i) −*x*+2, −*y*+1, −*z*+1.

### Hydrogen-bond geometry (Å, °)

**Table:**

*D*—H···*A*	*D*—H	H···*A*	*D*···*A*	*D*—H···*A*
N3—H3B···O1^i^	0.91	2.25	2.986 (3)	137
O1W—H1WA···O2^ii^	0.93	1.92	2.836 (4)	169
O1W—H1WB···O2^iii^	0.98	1.94	2.861 (3)	155
N2—H2B···O1W	0.86	1.86	2.706 (3)	168

Symmetry codes: (i) −*x*+2, −*y*+1, −*z*+1; (ii) −*x*+1, −*y*+1, −*z*+1; (iii) *x*, *y*+1, *z*+1.
